# Significance of abnormal blood coagulation in patients with chronic myelomonocytic leukemia

**DOI:** 10.1007/s10354-022-00969-4

**Published:** 2022-10-07

**Authors:** Christoph Weinfurtner, Klaus Geissler

**Affiliations:** 1 Medical School, Sigmund Freud University, Vienna, Austria; 2 Department of Internal Medicine V with Hematology, Oncology and Palliative Care, Hospital Hietzing, Wolkersbergenstraße 1, 1130 Vienna, Austria

**Keywords:** Chronic myelomonocytic leukemia, Prothrombin time, Survival, Coagulation, Prevalence, Chronische myelomonozytäre Leukämie, Prothrombinzeit, Überleben, Blutgerinnung, Prävalenz

## Abstract

In a retrospective study, we analyzed the prevalence of subnormal prothrombin time (PT) values in 104 patients with chronic myelomonocytic leukemia (CMML), their potential prognostic impact, and potential correlations with clinicolaboratory features. Reduced PT values (< 70%) were found in 45/104 (43%) patients. The median survival of patients with reduced PT values was significantly shorter than in patients with normal PT (19 vs. 49 months, *p* = 0.006). Patients with reduced PT had higher leukocyte counts, a higher proportion of circulating blast cells, and lower platelet counts. In patients for whom clinical information was available, there was no difference in the incidence of bleeding complications between patients with or without reduced PT. Our results show a high prevalence of plasmatic coagulation abnormalities in patients with CMML, which were associated with laboratory features of advanced disease. Moreover, subnormal PT values were identified as a new prognostic marker. Reduced PT values do not seem to have a clinical impact regarding bleeding complications.

## Abstract (de)

In einer retrospektiven Studie wurde die Prävalenz einer verminderten Prothrombinzeit (PTZ) bei 104 Patienten mit chronischer myelomonozytärer Leukämie (CMML), ihr potenzieller prognostischer Einfluss und ihre potenzielle Korrelation mit klinischen und laborchemischen Parametern untersucht. Eine verminderte PTZ (< 70 %) wurde bei 45/104 (43 %) Patienten gefunden. Das mediane Überleben von Patienten mit verminderten PTZ-Werten war signifikant kürzer als jenes von Patienten mit normalen PTZ-Werten (19 vs. 49 Monate, *p* = 0,006). Patienten mit verminderten PTZ-Werten wiesen höhere Leukozytenwerte, einen höheren Anteil zirkulierender Blasten und geringere Thrombozytenzahlen auf. Bei Patienten, bei denen klinische Informationen vorlagen, bestand kein Unterschied im Hinblick auf Blutungskomplikationen zwischen beiden Gruppen. Die vorliegenden Ergebnisse zeigen eine hohe Prävalenz plasmatischer Gerinnungsstörungen bei Patienten mit CMML, die mit Laborparametern einer fortgeschrittenen CMML assoziiert waren. Darüber hinaus wurde die PTZ als neuer prognostischer Parameter bei dieser Erkrankung identifiziert. Verminderte PTZ-Werte scheinen jedoch keinen Einfluss auf Blutungskomplikationen zu haben.

## Introduction

Chronic myelomonocytic leukemia (CMML) is a rare, genotypically and phenotypically heterogenous hematologic malignancy of elderly people, with an intrinsic risk of progression and transformation to secondary acute myeloid leukemia (AML). With regard to the presence of myeloproliferation, CMML was originally subdivided into myeloproliferative disorder (MP-CMML; white blood cell [WBC] count > 13 × 10^9^/L) versus myelodysplastic syndrome (MD-CMML; WBC count ≤ 13 × 10^9^/L MD-CMML) by the FAB criteria [1, 2]. Since CMML is characterized by features of both MDS and MPN, the World Health Organization (WHO) classification of 2002 assigned CMML to the mixed category, MDS/MPN [3]. CMML is further subclassified by the WHO into three groups based on blast equivalents (blasts plus promonocytes) in peripheral blood (PB) and bone marrow (BM) as follows: CMML‑0 if PB < 2% and BM < 5% blast equivalents; CMML‑1 if PB 2–4% or BM 5–9% blast equivalents; and CMML‑2 if PB 5–19% or BM 10–19% blast equivalents, and/or Auer rods are present [4]. CMML patients have a highly variable outcome, suggesting that several factors can determine the course of disease and the causes of death in these patients [5–9]. There are a number of established prognostic parameters that have been incorporated into several prognostic models [10–21]. The clinical significance of disturbed plasmatic coagulation in patients with CMML is poorly investigated. Prothrombin time (PT) is a widely used test of overall clotting function. Using the database of the Austrian Biodatabase for Chronic Myelomonocytic Leukemia (ABCMML), we analyzed 104 CMML patients with available information on PT values and other coagulation parameters [22]. This information from a real-life database could be useful in the management of these patients.

## Patients and methods

### Patients

Recently, we have shown that the ABCMML may be used as a representative and useful real-life data source for biomedical research [22]. In this database, we retrospectively collected epidemiologic, hematologic, biochemical, clinical, immunophenotypic, cytogenetic, molecular, and biologic data of patients with CMML from different centers. The diagnosis of CMML and leukemic transformation was according to the WHO criteria [2–4]. Clinical and laboratory routine parameters were obtained from patient records. A detailed central manual retrospective chart review was carried out to ensure data quality before analysis of data from institutions. Due to the fact that CMML may be considered as an evolutionary process from clonal hematopoiesis of indeterminate potential (CHIP) to CMML-related AML [23], and the fact that the distinction between mature and immature monocytic cells, which is required to determine the time of transformation into AML, is notoriously difficult due to the lack of reliable immunophenotypic markers, we found it more appropriate not to exclude the few CMML patients with transformation from our analysis [24].

For 104 CMML patients collected between 1.1.1990 and 31.3.2019, information was available regarding PT values. Patients on anticoagulation were not included in this study. This research was approved by the ethics committee of the City of Vienna on 10 June 2015 (ethic code: 15-059-VK).

### Statistical analysis

The log-rank test was used to determine whether individual parameters were associated with overall survival (OS). OS was defined as the time from sampling to death (uncensored) or last follow-up (censored). Multivariate Cox regression analysis of overall survival was used to describe the relation between the event incidence, as expressed by the hazard function, and a set of covariates. Dichotomous variables were compared between different groups using the chi-square test. The Mann–Whitney *U* test was used to compare two unmatched groups when continuous variables were nonnormally distributed. Results were considered significant at *p* < 0.05. Statistical analyses were performed with the SPSS v. 27 (IBM Corp., Armonk, NY, USA); the reported *p*-values are two-sided.

## Results

### Patient characteristics

The baseline characteristics of the 104 patients with CMML are shown in Table 1. In order to make comparisons with other published CMML cohorts possible, the percentages of patients regarding established prognostic parameters are given [17]. As seen in other CMML series, there was a male predominance among study patients and more than half of patients were aged 70 years or older [17]. The proportion of patients with leukocytosis > 13 G/L, anemia < 10 g/dL, thrombocytopenia < 100 G/L, and the presence of blast cells in peripheral blood (PB) was also comparable to other cohorts [17]. Four patients in this cohort have already transformed into CMML-related AML.

**Table 1:** Characteristics of chronic myelomonocytic leukemia patients

	Cases (*N* = 104)	Percent
*Age* Evaluable = 104		
< 70 years	34	33
≥ 70 years	70	67
*Sex* Evaluable = 104		
Male	75	72
Female	29	28
*Leukocytes* Evaluable = 104		
> 13 G/L	48	46
≤ 13 G/L	56	54
*Hemoglobin* Evaluable = 104		
< 10 g/dL	29	28
≥ 10 g/dL	75	72
*Platelets* Evaluable = 104		
< 100 G/L	40	38
≥ 100 G/L	64	62
*Peripheral blood blasts* Evaluable = 97		
Absent	65	67
Present	32	33

### Prevalence of blood coagulation abnormalities in CMML

Reduced PT values (< 70%) were found in 43/104 (41%) patients. The proportion of patients with hypofibrinogenemia (< 2 g/L) was 10/66 (15%). 2/36 (6%) patients with normal PT values had subnormal fibrinogen levels as compared to 8/30 (27%) patients with decreased PT values. Thus, the proportion of patients with hypofibrinogenemia was significantly higher in patients with reduced PT (*p* = 0.017).

### Correlation of decreased PT values with laboratory phenotype and clinical parameters

As shown in Table 2, CMML patients with decreased PT values significantly clustered with several adverse disease features, such higher leukocyte counts, a higher proportion of circulating blast cells, and lower platelet counts. There was a borderline association with lower hemoglobin levels. In patients for whom clinical information was available, there were 4/26 (15%) patients with bleeding complications in the group with normal PT values as compared to 2/15 (13%) in patients with subnormal PT levels. Thus, there was no difference in the proportion of patients with documented bleeding (*p* = 0.858).

**Table 2:** Laboratory features stratified by the presence or absence of decreased PT value

	All patients (*N* = 104)	PT < 70% (*n* = 43)	PT ≥ 70% (*n* = 61)	*P*-value
Age in years; median (range) Evaluable = 104	73 (44–92)	73 (52–90)	72 (44–92)	0.776
Sex (male); *n* (%) Evaluable = 104	74 (71%)	31 (72%)	43 (70%)	0.859
Leukocytes G/L; median (range) Evaluable = 104	12.3 (3.0–238)	17.8 (4.3–237)	10.8 (3.0–86.7)	0.001
Hemoglobin g/dL; median (range) Evaluable = 104	11.4 (4.3–16.5)	11.2 (4.3–15.3)	11.8 (6.2–16.5)	0.424
Platelets G/L; median (range) Evaluable = 104	130 (12–1148)	77 (12–608)	150 (23–1148)	0.000
PB blasts %; median (range) Evaluable = 94	0 (0–94)	0 (0–94)	0 (0–18)	0.011

*PT* prothrombin time, *PB* peripheral blood

### Impact of decreased PT values on survival

**Fig. 1:**
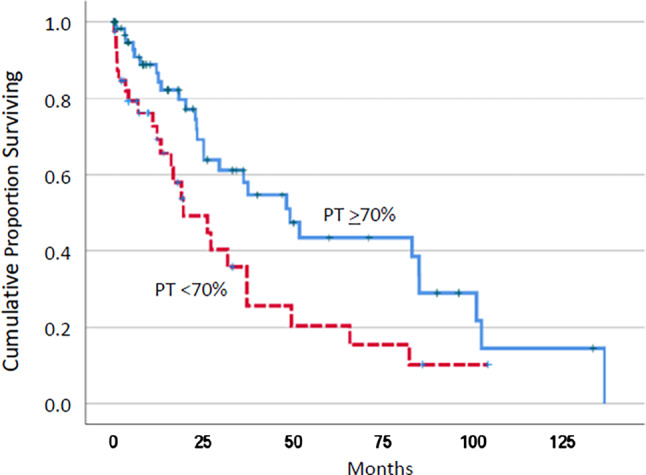
Kaplan–Meier plots for overall survival of chronic myelomonocytic leukemia patients with and without decreased prothrombin time (*PT*) values

As shown in Fig. 1, the median survival of patients with reduced PT values was significantly shorter than in patients with normal PT (19.3 vs. 49.0 months, *p* = 0.012). Table 3 shows the prognostic power of decreased PT values and established prognostic factors in the study population of CMML patients. As one can see, there was a significant adverse survival impact of all these factors, including leukocytosis > 13 G/L, anemia < 10 g/dL, thrombocytopenia < 100 G/L, and the presence of blast cells in PB. As shown in Table 4, the prognostic significance of PT values was lost in multivariate analysis in the presence of other adverse prognostic factors, indicating the dependence of coagulation abnormalities on parameters of advanced CMML.

**Table 3:** Univariate analysis of single prognostic parameters in patients with chronic myelomonocytic leukemia

Factors	Factor present Median OS (months)	Factor absent Median OS (months)	*P*-value (log-rank)
Decreased PT values	19.3	49.0	0.012
WBC > 13 × G/L	23.1	49.0	0.011
Hb < 10 g/dL	23.1	48.0	0.033
PLT < 100 × G/L	22.7	51.6	0.001
PB blasts present	18.8	37.3	0.002

The log-rank test was used to determine if individual parameters were associated with OS

*OS* overall survival, *WBC* white blood cell count, *Hb* hemoglobin, *PLT* platelet count, *PB* peripheral blood, *PT* prothrombin time

**Table 4:** Hazard ratios, confidence intervals, and *p*-values of Cox regression analysis for survival including decreased PT values and established prognostic parameters

Parameter	Hazard ratio	95% confidence interval	*P*-value
PT decreased (< 70%)	0.720	0.380–1.361	0.311
WBC ≥ 13 G/L	1.509	0.769–2.961	0.231
Hb < 10 g/dL	1.128	0.536–2.375	0.751
PLT < 100 × G/L	2.278	1.130–4.593	0.021
PB blasts present	1.735	0.900–3.346	0.100

*PT* prothrombin time, *WBC* white blood cell count, *Hb* hemoglobin, *PLT* platelet count, *PB* peripheral blood

### Coagulation abnormality in a patient with advanced CMML

In individual patients, disturbances of plasmatic coagulation were analyzed in more detail by the determination of single clotting factors. The analysis of one of these patients, who has already transformed into CMML-related AML, is shown in Table 5. As one can see, liver parameters, including bilirubin, ASAT, ALAT, gamma-GT, and alkaline phosphatase, were increased, whereas cholinesterase was decreased, indicating liver cell damage. Regarding clotting factors, all of them, excluding factor VIII, were decreased, suggesting compromised synthesis of these factors in the liver as opposed to disseminating intravascular coagulation in which all factors, including factor VIII, are typically decreased.

**Table 5:** Liver and coagulation parameters in a single patient with leukemia-associated liver failure

Parameter	Result	Normal values
ASAT	56 U/L	< 31 U/L
ALAT	51 U/L	< 34 U/L
Gamma-GT	99 U/L	< 38 U/L
Alkaline phosphatase	236 U/L	< 104 U/L
Cholinesterase	1146 U/L	4000–12 000 U/L
Bilirubin	1.98 mg/dL	0.2–1.2 mg/dL
LDH	1309 U/L	125–243 U/L
PT	44%	70–130%
aPTT	50.4 s	23–37 s
Fibrinogen	1.17 g/L	2.13–3.93 g/L
D‑dimer	1.9 mg/L	< 0.5 mg/L
ATIII activity	20%	80–130%
Factor II activity	25%	79–131%
Factor V activity	46%	75–130%
Factor VII activity	19%	75–160%
Factor X activity	50%	70–150%
Factor VIII activity	113%	60–160%
Factor IX activity	49%	60–140%
Factor XI activity	34%	60–140%
Factor XII activity	23%	60–140%
Factor XIII activity	62%	70–140%

*PT* prothrombin time, *aPTT* activated partial thromboplastin time

## Discussion

Abnormalities of plasmatic coagulation in CMML were described in 1979 in a few patients. In a series of 9 patients, 7 had abnormal coagulation values; in 2 cases, abnormalities were consistent with disseminated intravascular coagulation that correlated with hemorrhagic diathesis [25]. Cells of the human myelomonocytic line RC-2A synthesize tissue factor-like procoagulant and urokinase-type plasminogen activator [26]. Overall, the clinical significance of disturbed plasmatic coagulation abnormalities in patients with CMML is poorly investigated.

In this paper, we show that decreased PT values are an adverse prognostic parameter in CMML. This finding is new and has not been, to the best of our knowledge, reported by others. Interestingly, decreased PT values were not associated with significant bleeding tendency, including the incidence of major bleeding. It has to be noted, however, that the number of CMML patients with information regarding bleeding complications was low. By correlating PT values with laboratory parameters, we found higher leukocyte counts, a higher proportion of circulating blast cells, and lower platelet counts in CMML patients with decreased PT. Moreover, we found that the prognostic significance of PT values was lost in the presence of established prognostic factors such as leukocytosis, anemia, thrombocytopenia, and circulating blasts in multivariate analysis. Altogether, these results suggest that blood coagulation abnormalities in CMML patients may not be an independent prognostic factor but may be associated with more advanced disease. Since blood coagulation parameters are synthesized in the liver, one could speculate that blood coagulation abnormalities may be due to infiltration of the liver by CMML cells. Indeed, it has been shown that in a subgroup of patients with CMML, hepatomegaly can be demonstrated [27]. Moreover, we have seen a correlation of decreased PT values with increased liver parameters, and could demonstrate in single patients with detailed analysis of coagulation factors a reduction of coagulation factors excluding factor VIII, which is a characteristic pattern for impaired liver synthesis. There were 2 patients with subnormal fibrinogen levels and normal PT values. Although not analyzed, one can speculate that in these patients, increased production of urokinase-type plasminogen activator may have contributed to this finding, as was proposed in a previous study [26].

We are aware of the limitations of our study. For example, most of the information used in this study was derived from retrospective real-world data that were not collected systematically or prospectively. Thus, not every parameter was available in all patients. In addition, data from patient records were obtained over many years and from many different centers. Moreover, the patients included in this study represented a relatively heterogenous population regarding the blast cell counts. However, real-world data have recently been recognized as an important way to get insights into routine management and the natural history of rare diseases [28]. CMML is a rare disease, and adequate patient numbers for a systematic and prospective study are not easy to collect within a limited timeframe. Moreover, the ABCMML provides information derived from molecular as well as from functional studies, and therefore allows a more comprehensive view and deeper insight into the complex pathophysiology of this hematologic malignancy [22].
